# Regulation of RNA methylation linked to drug resistance in gastric cancer

**DOI:** 10.3389/fcell.2025.1686025

**Published:** 2026-01-08

**Authors:** Zihan Yang, Yanpin Ma, Ziyi Xu, Hongyan Liu, Xinyu Gu, Jiachun Sun

**Affiliations:** Henan Key Laboratory of Cancer Epigenetics, Cancer Institute, The First Affiliated Hospital, College of Clinical Medicine, Medical College of Henan University of Science and Technology, Luoyang, China

**Keywords:** RNA methylation, GC, drug resistance, epigenetic modification, immunotherapy

## Abstract

In global terms, gastric cancer (GC) represents one of the most commonly occurring malignancies. It is positioned as the fifth most frequent cancer in terms of incidence and stands as the third primary contributor to cancer-related mortality. As per the latest global cancer report from 2020, there were approximately 1.1 million new cases of GC and about 800,000 new deaths in that year, making up 5.6% of new cases and 7.7% of deaths related to cancer. In recent years, as bioinformatics technology and high-throughput sequencing have advanced rapidly, our comprehension of the genetic and epigenetic alterations associated with GC has also progressed considerably. Among these alterations, RNA methylation, as one of the common modifications within RNA molecules, has been regarded as a key factor in the development and progression of GC. Research indicates that the dysregulation of RNA methylation influences GC development through various pathways. Therefore, understanding the pathogenic mechanisms of RNA methylation in GC is of great significance for the diagnosis, treatment and prognostic assessment of affected patients. In this review, we discuss various types of RNA methylation, including N6-methyladenosine (m6A), 5-methylcytosine (m5C), N7-methylguanosine (m7G), and N1-methyladenosine (m1A), and how they might affect the mechanism of GC. We also look at how RNA methylation impacts chemotherapy, targeted therapy, and immune resistance in gastric cancer, as well as the potential uses of RNA methylation in treating gastric cancer, setting the stage for more detailed research on RNA methylation in gastric cancer.

## 1 Introduction

GC is a major global health concern and the fifth most prevalent malignancy (Smyth et al., 2020). Anatomically, GC can be categorized into the following two sub-types: cardia gastric cancer (CGC) and non-cardia gastric cancer (NCGC). NCGC stands for Non-Cardia Gastric Cancer. It refers to cancer occurring in the part of the stomach that’s not the cardia, usually found in areas like the fundus, body, and antrum (Li X. et al., 2024). Since most GC cases are diagnosed at an advanced stage, the mortality rate is high, making GC the third most common cause of cancer-related deaths. Among the multiple causes of GC, infection by the bacterium *Helicobacter pylori* is the most common risk factor for NCGC. Other risk factors for NCGC include advanced age, low socioeconomic status, smoking, alcohol consumption, familial history, a history of gastric surgery, malignant anemia, and a history of residing in high-risk population areas (Smyth et al., 2020). Despite the significant progress in GC treatment over the past few years (including endoscopic treatment, surgical resection, targeted therapy, immunotherapy, and chemotherapy), the outlook for GC patients is still poor; especially, the 5-year survival rate for patients with advanced cancer is still below 5% (Quan et al., 2025). Although chemotherapy serves as the primary therapy for advanced GC, its efficacy is often limited due to the common occurrence of drug resistance (Khaleel et al., 2024). The relevant mechanisms can be quite complicated, involving multiple aspects such as drug targets, apoptosis, autophagy, and the tumor immune microenvironment. Thus, chemotherapy resistance as a common issue in GC cancer is a great hurdle that directly impacts patient survival and quality of life (Shi and Gao, 2016).

Epigenetics studies reversible and inheritable phenotypes, encompassing aspects such as DNA and RNA methylation, histone modifications, chromatin rearrangement, and the role of non-coding RNA (ncRNA) modifications. Epigenetic modifications refer to alterations after the translation of heritable genes without changing the DNA sequence, primarily involving histone and nucleic acid modifications. RNA modifications, an important branch of epigenetics, include over 100 types identified so far (Dunin-Horkawicz et al., 2006). Of these modifications, RNA methylation constitutes over 60% of all known types. RNA methylation exists in various forms and significantly contributes to the regulation of multiple facets of RNA processing, encompassing RNA transcriptome management, splicing and exportation (Bao et al., 2024). RNA methylation is involved in cancer development, spread and resistance to treatment by regulating the expression of key oncogenes and tumor suppressor genes; therefore, its regulating enzymes have become new targets for therapy, while they also hold potential as cancer diagnostic and prognostic biomarkers (Yang B. et al., 2021; Li Y. et al., 2024; Han et al., 2023).

Evidence suggests a significant association between epigenetic alterations and the advancement of GC. For example, the Methyltransferase-like 3 (METTL3)-Insulin-like Growth Factor 2 mRNA-Binding Protein 3 (IGF2BP3)-m6A axis enhances angiogenesis, glycolysis and hypoxic adaptation in GC by stabilizing hepatoma-derived growth factor (HDGF) and Hypoxia-Inducible Factor 1 Alpha (HIF1A) mRNA, thereby driving tumor progression and metastasis (Han et al., 2023). In the past decade, with the increasing application of epigenetic drugs in clinical settings, the role of epigenetics in GC has become increasingly prominent (Hudler, 2018; Grady et al., 2021). Research indicates that epigenetic modifications in GC significantly contribute to the processes of cancer initiation, progression, diagnosis, and therapeutic strategies, even appearing earlier than gene mutations, indicating great potential for early diagnosis and targeted therapy (Yang Q. et al., 2021; Zeng et al., 2017; Sogutlu et al., 2022). Therefore, conducting in-depth epigenetic analyses of GC can provide unique insights into the pathogenesis of GC and molecular targeted therapies. In this review, we comprehensively discuss the relevant aspects of RNA methylation in GC, including RNA methylation levels, functions, and their relationship with GC drug resistance mechanisms, grounded in the most recent research findings. Furthermore, beyond the molecular mechanisms, we also detail the prospective clinical applications of RNA methylation in GC from both prognostic and therapeutic viewpoints.

## 2 RNA methylation

**FIGURE 1:**
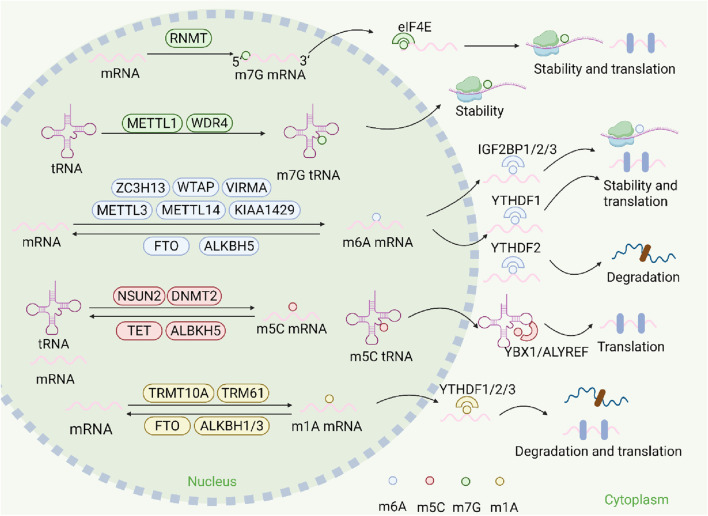
The mechanism of RNA methylation. RNA methylations are modulated by their writers (such as METTL3/14 for m6A, NSUN2 for m5C, TRMT10A for m1A, METTL1 for m7G), and removed by their erasers (such as FTO and ALKBH5for m6A). RNA methylations can regulate the fates of mRNA and mediate their biological functions including splicing, exportation, stability, degradation, translation and so on, after being recognized by their respective readers, including IGF2BP1/2/3, YTHDF1/2/3, YBX1,ALYREF, eIF4E).

A prevalent modification that is present across various RNA types is RNA methylation, encompassing messenger RNA (mRNA), transfer RNA (tRNA), microRNAs (miRNAs), ribosomal RNA (rRNA), as well as ncRNA. These alterations play a significant role in overseeing multiple facets of targeted RNA processing, including the processing of the RNA transcriptome, splicing mechanisms, and exportation (Bao et al., 2024). RNA methylation mainly refers to m6A, m5C, m7G, m1A, etc. Different types of RNA methylation can regulate the structure and function of RNA, thereby affecting gene expression and cellular fate, playing an important role in the occurrence and development of diseases such as cancer (Su et al., 2019; Zhao et al., 2021). RNA methylation affects the translation and stability of RNA through specific regulatory proteins, including methyltransferases (writers), demethylases (erasers), and methylation reading proteins (readers), which in turn affect the imbalances of immune cells and immune factors (Li Y. et al., 2024; Zeng et al., 2024) (Figure 1). Within this context, writers play a role in introducing modifications, while erasers are tasked with the removal of these modifications. Additionally, binding proteins are essential for the recognition of these modifications, subsequently influencing associated biological processes. These three components work together to influence various biological processes in cells, with this influence varying across different cellular environments. These processes give important directions for research in related fields (Chen D. et al., 2024).

### 2.1 m6A methylation

A well-established modification of RNA found in eukaryotic messenger RNA (mRNA) is N6-methyladenosine (m6A) methylation, which has various influences such as on RNA stability, splicing processes, and the efficiency of translation (Wang Z. et al., 2023). M6A is mainly regulated by writers, erasers and methylation reader proteins. The m6A methylation process is facilitated by several writers, specifically Methyltransferase-like 3 (METTL3), Methyltransferase-like 14 (METTL14), Wilms Tumor 1-Associating Protein (WTAP), RNA-Binding Motif Protein 15/15B (RBM15/15B), Zinc Finger CCCH-Type Containing 13 (ZC3H13), and Vir-like m6A Methyltransferase Associated (KIAA1429), among which METTL3, METTL14 and WTAP together form the m6A methyltransferase complex (MTC). Research indicates that the interaction between METTL3 and METTL14 is crucial for facilitating the m6A modification. Furthermore, WTAP acts as a regulatory component, promotes the identification of particular RNA molecules by the complex, consequently improving the efficacy of m6A modification (Jansens et al., 2022). The primary m6A erasers consist of Fat Mass and Obesity-Associated Protein (FTO) and Alkylation Repair Homolog 5 (ALKBH5). These enzymes play a critical role in modulating RNA stability and functionality through the removal of m6A modifications. The distinct catalytic mechanisms of FTO and ALKBH5 have been established by numerous studies. In particular, FTO is capable of demethylating m6A and transforming it into either N6-hydroxymethyladenosine (hm6A) or adenosine (A), whereas ALKBH5 directly converts m6A into A (Feng et al., 2021). The activity of these two enzymes is crucial for maintaining the dynamic balance of m6A levels within cells. m6A readers can specifically bind to m6A modification sites, thereby affecting RNA stability, translation efficiency and degradation, playing a key role in the recognition and regulation of m6A modifications. The principal classification of m6A readers is as follows: YTH domain family proteins 1/2/3 (YTHDF1/2/3), insulin-like growth factor 2 mRNA-binding proteins 1/2/3 (IGF2BP1/2/3), HNRNP family proteins, eukaryotic translation initiation factor 3 (eIF3), and eukaryotic translation initiation factor 4E (eIF4E) (Wang et al., 2023). YTHDF1, YTHDF2 and YTH Domain Containing 1 (YTHDC1) are typical m6A binding proteins: YTHDF1 facilitates the translation process of m6A-modified messenger RNA, whereas YTHDF2 is involved in the degradation of m6A-modified messenger RNA (Yang D. et al., 2023). IGF2BP family proteins enhance the stability of m6A-modified RNA and promote translation, playing important roles in tumor occurrence and development (Li R. et al., 2024). HNRNP family proteins also participate in the regulation of m6A modifications, affecting RNA splicing and transport (Cui S. et al., 2024). Through these mechanisms, m6A-binding proteins significantly contribute to various biological processes, including cellular proliferation, differentiation, and the development of tumors. Studies have shown that m6A modifications play significant roles in RNA splicing, transport, stability, and translation efficiency, consequently influencing a range of cellular biological processes (Li G. et al., 2024). Simultaneously, the concentration and localization of m6A exhibit considerable differences among various cell types and physiological conditions. This variability positions m6A as a key biomarker and therapeutic target in cancer research, investigations into immune responses, and other related studies (Xu and Shen, 2022).

### 2.2 m5C methylation

5-Methylcytosine (m5C) represents a noteworthy methylation modification of RNA, characterized by the addition of a methyl (-CH_3_) group to the carbon at the five-position of cytosine. This modification is extensively found in various types of RNA, including mRNA, tRNA and rRNA (Chen Y. S. et al., 2021). The biological roles of m5C predominantly revolve around the modulation of RNA stability, enhancement of translation efficiency, and determination of intracellular localization (Wu et al., 2025). M5C exerts a considerable influence over a range of biological processes, such as cellular proliferation, differentiation, migration, and apoptosis (Li Y. et al., 2024). Similar to m6A modifications, m5C modifications are also reliant on a distinct set of enzymes known as writers and erasers. The writers responsible for m5C include members of the NOP2/Sun RNA Methyltransferase family (NSUN) and DNA Methyltransferase 2 (DNMT2). These enzymes play a crucial role in modulating RNA stability and translation by facilitating the transfer of methyl groups to cytosine residues present in RNA molecules (Abbas et al., 2024; Zhou et al., 2020). The enzymes known as erasers for m5C comprise the Ten-Eleven Translocation (TET) family and the ALKBH family. These facilitate the conversion of m5C into alternative chemical forms, subsequently influencing both the stability and functionality of RNA (Simpson et al., 2023). For instance, TET enzymes are not only involved in the process of DNA demethylation but have also been shown to eliminate m5C modifications from RNA, thereby influencing both RNA translation and degradation (Yang et al., 2024). Additionally, a complex regulatory network exists between m5C erasers and writers, forming a dynamic balance system that regulates the methylation status of RNA, thereby affecting the biological functions of cells and the development of tumors (Cui et al., 2025). At the same time, the biological roles of m5C modifications are modulated by particular binding proteins, including Y-box binding protein 1 (YBX1) and Aly/REF nuclear export factor (ALYREF). These proteins possess the capability to identify m5C alterations, thereby affecting both RNA stability and translation efficiency (Cui Y. et al., 2024). In conclusion, the mechanism of m5C modification plays a crucial role in the regulation of gene expression and the functioning of cells.

### 2.3 m7G methylation

N7-methylguanosine (m7G) predominantly exists in the mRNA, tRNA and miRNA of eukaryotic organisms. The chemical architecture of this compound is defined by the incorporation of a methyl group at the 7-position of guanosine, resulting in the creation of a distinctive cap structure located at the 5′terminal of RNA (Luo et al., 2022). m7G modifications have been shown to play a significant role in numerous biological processes by influencing the efficiency of RNA translation, splicing mechanisms, and the exportation of RNA from the nucleus (Chen Z. et al., 2021). m7G modifications depend on specific writers and binding proteins. The primary methyltransferase responsible for the addition of the m7G modification is the METTL1/WD Repeat Domain 4 (WDR4) complex. Therein, METTL1 facilitates the transfer of methyl groups, while WDR4 plays a crucial role in augmenting the catalytic activity and substrate specificity of METTL1. Collectively, this complex introduces m7G modifications onto tRNA, mRNA and cap structures, thereby contributing to the regulation of RNA stability and translation efficiency (Zhou et al., 2024; Li et al., 2023). The proteins that bind to m7G comprise components of the cap-binding complex, such as eIF4E. Via identifying m7G caps or specific internal sites, these facilitate the export of mRNA from the nucleus, initiate translation, and ensure effective interaction with ribosomes (Osborne et al., 2022; Haimov et al., 2018). Moreover, m7G binding proteins establish intricate regulatory networks via their interactions with various RNA-binding proteins, thereby influencing RNA metabolism and its functional roles (Han et al., 2024). Consequently, understanding the function of m7G modifications along with their associated regulatory proteins in cellular activities holds considerable importance.

### 2.4 m1A methylation

N1-methyladenosine (m1A) represents a modification of RNA that influences both gene expression and the stability of RNA molecules. This modification is predominantly observed in tRNA and mRNA and is defined by the insertion of a methyl group at the 1-position of the adenosine nucleotide (Peer et al., 2017). In a manner akin to m6A modifications, m1A is chiefly governed by three distinct categories of regulatory proteins: “erasers” such as ALKBH1, ALKBH3 and FTO, “writers” including TRMT10C, TRMT61B and TRMT6/61A, and “readers” comprising YTHDF1, YTHDF2, YTHDF3 and YTHDC1. In particular, the enzymes TRMT61B, TRMT6/61 and TRMT10C facilitate the addition of m1A modifications at various locations on mitochondrial RNA within human cells. Concurrently, proteins from the YTHD family are capable of identifying m1A modifications, thereby influencing subsequent RNA translation and degradation processes. Furthermore, the ALKB family members, specifically ALKBH3 and ALKBH1, possess the ability to excise m1A from both single-stranded DNA and RNA molecules (Zhang C. et al., 2023). Various studies have indicated that m1A plays a role in modulating local structural integrity, interactions between RNA and proteins, cellular apoptosis, and the process of cell proliferation (Li et al., 2021). For instance, the m1A modification can facilitate the proper folding and functionality of tRNA, which in turn influences the process of protein synthesis (Jin et al., 2022). Moreover, alterations in m1A methylation levels are strongly associated with the initiation and progression of tumors, positioning it as a promising target for therapeutic intervention (Su et al., 2022a). Thus, comprehensive investigations into the mechanisms underlying m1A modifications, along with the roles of their associated regulatory proteins, hold considerable importance for elucidating RNA biology and its implications in various diseases.

## 3 Expression characteristics and clinical significance of RNA methylation in GC

**FIGURE 2:**
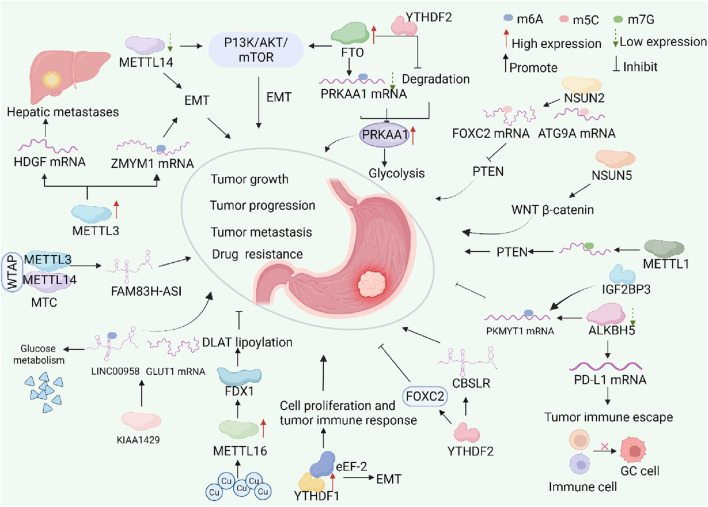
The dynamic process of RNA modifications in GC and their potential molecular mechanisms. Abnormal RNA methylation is prevalent in most stages of gastric cancer. RNA methylation is a dynamic modification that involves m6A methyltransferases, demethylases, and methylation-binding proteins. Abnormal expression of RNA methylation regulators often impacts downstream genes and signaling pathways. This involvement plays a role in the onset and progression of gastric cancer. Tumor growth is when tumor cells grow in size through proliferation, escaping apoptosis, and growing locally, while tumor progression is when the tumor becomes more malignant, showing broader biological behaviors such as invasion, metastasis, angiogenesis, and resistance to treatment.

In recent years, a multitude of investigations have highlighted that RNA methylation is instrumental in the development of gastrointestinal cancers, with a particular emphasis on GC, thereby highlighting its considerable clinical relevance (Li G. et al., 2022; Wu et al., 2022; Lu et al., 2025). Similar to DNA methylation and histone modifications, RNA methylation can affect the expression of key oncogenes or tumor suppressor genes by regulating RNA stability, splicing, translation, and localization, thus playing a role in the onset, progression and treatment resistance of GC (Shen et al., 2020) (Figure 2). Clinical studies have shown that there are abnormalities in RNA methylation levels in GC tissues, which are closely related to the tumor’s TNM staging, lymph node metastasis, and chemotherapy resistance (Su et al., 2019; Zhao et al., 2021).

### 3.1 Molecular mechanisms of m6A in GC initiation and progression

In GC, there is a notable alteration in the expression levels of several enzymes associated with RNA methylation. (Table 1). m6A represents the predominant RNA modification observed in GC. A significant proportion of patients diagnosed with GC exhibit elevated expression levels of m6A methylation regulatory factors within their tumor tissues, a phenomenon that is intricately linked to the malignancy level of the tumor (Su et al., 2019; Zhao et al., 2021; Xu et al., 2022).

**TABLE 1:** The role and mechanism of RNA methylation in GC.

Methylations	Type	Regulator of RNA methylation	Expression	Functions in GC	Related targets	Year	Reference
m6A	Writer	METTL14	Down	Promote the proliferation and invasion ability of gastric cancer cells	PIK3, AKT, mTOR	2019	Yue et al. (2019)
	Writer	METTL14	Down	Cell viability, colony formation, and cell invasion, Tumor growth	circORC5, miR-30c-2-3p, and AKT1S1	2022	Wang et al. (2020)
	Writer	METTL3	Upregulation	Methylations	P300, H3K27, IGF2BP3, HDGF, GLUT4, and ENO2	2023	Hudler (2018)
	Writer	METTL3	Upregulation	Cell invasion, and cell migration Lung metastasis, and liver metastasis	ZMYM1, CtBP/LSD1/CoRESTand E-cadherin	2019	Zhang Z. et al. (2023)
	Writer	METTL3	Upregulation	Cell proliferation, and cell migration Tumor growth, and metastasis	IGF2BP2, STAT5A, and KLF4	2024	Yu and Yang (2024)
	Writer	METTL16	Upregulation	Promote copper-mediated cell death in gastric cancer cells	FDX1	2023	Liu and Da (2023)
	Writer	WTAP	Upregulation	Promote the proliferation, migration, invasion and drug resistance of gastric cancer cells	—	2024	Zhang et al. (2019)
	Writer	KIAA1429	Down	Promote glucose metabolism in gastric cancer cells and the progression of malignancy	LINC00958	2021	Sun et al. (2023)
	Eraser	FTO	Down	Promote the proliferation and glycolysis of gastric cancer cells	PRKAAI	2022	Yang B. et al. (2021)
	Eraser	FTO	Upregulation	Promote the migration, invasion and proliferation of gastric cancer cells and the EMT process	PIK3、AKT	2023	Zhang et al. (2022a)
	Eraser	ALKBH5	Upregulation	Cell migration, and cell invasion	NEAT1, and EZH2	2019	Zhu et al. (2023)
	Eraser	ALKBH5	Upregulation	Promote the migration, invasion and proliferation of gastric cancer cells and tumor growth	LINC00659, YTHDF2, and JAK1	2023	Hu et al. (2022)
	Eraser	ALKBH5	Down	Inhibiting the invasion and metastasis of gastric cancer	PKMYT1	2022	Qiu et al. (2025)
	Eraser	ALKBH5	Down	Mediating tumor immune escape	—	2025	Jiang et al. (2024)
	Reader	IGF2BP1	Upregulation	Related to the poor prognosis of the patient	—	2024	Liu et al. (2020)
	Reader	YTHDF1	Upregulation	Induction of metastasis of gastric cancer cells and the process of EMT	eEF-2	2020	Yang et al. (2022)
	Reader	YTHDF2	Upregulation	Inhibit cell proliferation	FOXC2	2023	Hu et al. (2021)
	Reader	YBX1	Upregulation	Promoting gastric cancer proliferation, 5-FU resistance, and autophagy	ATG9A	2025	Fatima (2025)
m5C	Writer	NSUN2	Upregulation	Promote the proliferation of gastric cancer cells, the progression of the G1/S phase of the cell cycle, and the *in vivo* tumor-forming ability	p57Kip2	2020	Yan et al. (2021)
	Writer	NSUN2	Upregulation	Promoting the migration, invasion and nerve invasion (NI) of gastric cancer cells is associated with poor prognosis	NTN1	2023	Liu S. et al. (2024)
	Writer	NSUN2	Upregulation	Promote the proliferation, migration and invasion of gastric cancer cells	SUMO-2/3	2021	Li H. et al. (2022)
	Writer	NSUN2	Upregulation	Promote the proliferation, migration, invasion and drug resistance of gastric cancer	FOXC2、ATG9A、PTEN	2021	Christodoulidis et al. (2024)
	Writer	NSUN5	Upregulation	Promote the proliferation and migration of gastric cancer cells	Cyclin D1、c-MYC	2024	Yue et al. (2023)
m7G	Writer	METTL1	Upregulation	Promote the progression of gastric cancer and reduce the immune response	—	2022	Pi et al. (2021)

In the context of m6A modification, elevated levels of METTL3 expression correlate with unfavorable prognosis, increased tumor malignancy, and reduced overall survival (OS) (Yu and Yang, 2024; Zhang Z. et al., 2023; Su et al., 2022b). METTL3 increases the stability of ZMYM1 mRNA through the catalysis of its m6A modification, which facilitates the process of epithelial-mesenchymal transition (EMT) and thus enhances the invasion and metastasis of GC cells (Yue et al., 2019). Additionally, METTL3 can regulate the stability of HDGF mRNA through m6A methylation, promoting tumor growth and liver metastasis in GC (Wang et al., 2020). The above findings indicate that METTL3 could represent a promising therapeutic target for GC. In GC tissues, reduced levels of METTL14 lead to a decrease in m6A modifications, which subsequently activates the PI3K/AKT/mTOR signaling pathway as well as the EMT pathway. This biochemical alteration in turn facilitates the proliferation and invasive capabilities of GC cells (Yang P. et al., 2023; Zhang et al., 2019). Studies have indicated that WTAP exhibits elevated expression levels in GC tissues, functioning as a pivotal “connector” within the m6A methylation pathway. This role of WTAP facilitates the stabilization of the METTL3/METTL14 complex. Furthermore, WTAP enhances the proliferation, migration and invasion capabilities of GC cells by mediating the m6A modification of the long noncoding RNA (lncRNA) FAM83H-AS1 (Liu N. et al., 2024). WTAP has the capacity to affect the EMT mechanism in GC as well as the resistance of GC cells to therapeutic agents (Liu and Da, 2023). Research has demonstrated that the levels of copper ions in GC tissues are markedly elevated when compared to those found in normal tissues. Under copper stress, the m6A methyltransferase METTL16 undergoes lactylation modification, enhancing its activity. When the activity of METTL16 is increased, this stabilizes the upregulation of FDX1 protein expression through m6A modification, inducing DLAT acylation, ultimately triggering copper death in GC cells, a finding that provides new diagnostic markers and therapeutic targets for GC (63). KIAA1429 can catalyze m6A modifications on LINC00958 and mediate its interaction with GLUT1 mRNA, increasing the stability of GLUT1 mRNA, promoting glucose metabolism and malignant progression in GC cells (Yang D. et al., 2021).

FTO reduces the m6A modification of PRKAA1 mRNA through demethylation, inhibiting YTHDF2-mediated degradation and thereby upregulating PRKAA1 expression. FTO is positively correlated with PRKAA1 levels, both promoting the proliferation and glycolysis of GC cells (Zhang et al., 2022a). Research has indicated that elevated levels of FTO expression are closely linked to unfavorable outcomes in patients with GC. This elevated expression stimulates the PI3K/AKT/mTOR signaling cascade, thereby enhancing the migration, invasion and proliferation of GC cells, as well as facilitating the EMT process (Zhu et al., 2023). ALKBH5 is downregulated in GC tissues and is associated with the distant metastasis and lymph node metastasis of tumors. Also, it regulates the stability of PKMYT1 mRNA mediated by IGF2BP3 in an m6A-dependent manner, thus inhibiting the invasion and metastasis of GC (Hu et al., 2022). Besides, the downregulation of ALKBH5 can stabilize PD-L1 mRNA, mediating tumor immune evasion (Qiu et al., 2025). Elevated concentrations of IGF2BP1 have been linked to the immune response and associated with unfavorable outcomes in individuals diagnosed with GC (Jiang et al., 2024).

YTHDF1 exhibits elevated expression in GC, which facilitates the progression of this malignancy by influencing cellular proliferation and the immune response within the tumor microenvironment. YTHDF1 cooperates with eEF-2 to induce the migration and EMT process of GC cells (Liu et al., 2020); meanwhile, YTHDF2 enhances ferroptosis tolerance via CBSLR and inhibits cell proliferation through FOXC2 signaling, indicating its dual potential of both pro-cancer and anti-cancer and the potential to serve as a prognostic indicator (Yang et al., 2022).

### 3.2 Roles of other types of RNA methylation in GC

Beyond m6A, there has been a focus on other RNA modification-associated genes to assess their potential diagnostic and prognostic significance in GC. For instance, the m5C methyltransferase NSUN2 exhibits a marked overexpression in GC tissues and is strongly associated with unfavorable patient outcomes. It facilitates the proliferation, migration and invasion of GC cells through m5C modifications and the SUMO-2/3-mediated localization within the nucleus (Hu et al., 2021). Additionally, NSUN2 increases mRNA stability (FOXC2, ATG9A) through the catalysis of m5C modification. It also reduces PTEN expression and promotes the growth, movement, invasion, and resistance to drugs in GC (Yan et al., 2021). NSUN5 enhances the growth and movement of GC cells through the activation of the WNT/β-catenin signaling cascade (Liu S. et al., 2024). Similarly, the m7G methyltransferase METTL1 enhances mRNA translation efficiency (such as genes related to the PTEN pathway) through catalyzing m7G modifications, regulates the immune microenvironment (inhibiting Th1/Th2/CD8+ cell infiltration), suppresses tumor suppressor gene functions, and promotes GC progression, reducing immune responses and worsening prognosis (Li X. Y. et al., 2022). However, research on the links between m1A methylation-related factors and GC have been scarce.

### 3.3 Application prospects of RNA methylation in GC

Studies have extensively explored the interaction between RNA methylation and GC progression, establishing the significant promise of targeted RNA methylation both *in vitro* and in various animal models (Table 2). RNA methylation, especially the m6A modification, is considered a promising biomarker for both diagnosis and prognosis in GC. Changes in m6A methylation levels can serve as biomarkers for the early diagnosis of GC, especially in liquid biopsies where RNA molecules exhibit high specificity and sensitivity (Liu et al., 2020; Christodoulidis et al., 2024). For example, m6A-related risk scoring models can effectively distinguish high-risk from low-risk GC patients and predict their prognosis (Yue et al., 2023). Mechanistically, RNA methylation facilitates the proliferation, epithelial-mesenchymal transition and resistance of GC cells to chemotherapy through the modulation of critical signaling pathways, including Wnt/β-catenin, PI3K/AKT and NF-κB (Wang H. et al., 2024; Pi et al., 2021). Animal model experiments further demonstrated that inhibitors targeting METTL3 or NSUN2 (such as STM2457) can significantly slow down the progression of GC. This finding offers substantial clinical evidence to successfully employ targeted therapies focusing on RNA methylation (Fang et al., 2025; Li et al., 2025). In summary, the detection of RNA methylation not only aids in the early diagnosis of GC but also provides important biomarkers for clinical prognosis assessment, providing new options for the early diagnosis, prognostic evaluation, and precise treatment of GC.

**TABLE 2:** Clinical applications of RNA methylation in GC.

Methylations	Type	Effects	Clinical implication	Relevant medications	Regulator of RNA methylation	Year	Reference
m6A	Methyltransferase	Tumor promoter	Prognosis	—	METTL3	2019	Zhang Z. et al. (2023)
	Methyltransferase	Tumor promoter	Prognosis and Treatment	STM2457 and PD-L1 monoclonal antibody	METTL3	2024	Han et al. (2023)
	Methyltransferase	Tumor promoter	Prognosis and Treatment	Oxaliplatin	METTL3	2022	Chen et al. (2022)
	Methyltransferase	Tumor suppressor	Prognosis and Treatment	Cisplatin	METTL14	2024	Li X. Y. et al. (2022)
	Methyltransferase	Tumor suppressor	Prognosis	—	METTL14	2019	Yue et al. (2019)
	Methyltransferase	Tumor promoter	Prognosis	—	WTAP	2024	Zhang et al. (2019)
	Methyltransferase	Tumor promoter	Prognosis and Treatment	Elesclomol and AGK2	METTL16	2023	Liu and Da (2023)
	Methyltransferase	Tumor promoter	Prognosis and Treatment	Cisplatin	KIAA1429	2022	Chen X. Y. et al. (2024)
	Methyltransferase	Tumor promoter	Prognosis and Treatment	Oxaliplatin	KIAA1429	2023	Song et al. (2025)
	Methyltransferase	Tumor promoter	Prognosis	—	KIAA1429	2021	Sun et al. (2023)
	Demethylase	Tumor promoter	Prognosis	​	ALKBH5	2019	Zhu et al. (2023)
	Demethylase	Tumor promoter	Prognosis	​	ALKBH5	2023	Hu et al. (2022)
	Demethylase	Tumor suppressor	Prognosis	—	ALKBH5	2025	Jiang et al. (2024)
	Demethylase	Tumor suppressor	Prognosis	—	ALKBH5	2022	Qiu et al. (2025)
	Demethylase	Tumor promoter	Prognosis	—	FTO	2023	Zhang et al. (2022a)
	Binding proteins	Tumor promoter	Prognosis and Treatment	Cisplatin	YTHDF1	2022	Tang et al. (2023)
	Binding proteins	Tumor promoter	Prognosis	Oxaliplatin	YTHDF2	2025	Zhu Z. et al. (2022)
	Binding proteins	Tumor promoter	Prognosis	—	IGF2BP3	2022	Qiu et al. (2025)
	Binding proteins	Tumor promoter	Prognosis	PD-1 monoclonal antibody	IGF2BP3	2024	Han et al. (2023)
	Binding proteins	Tumor promoter	Prognosis and Treatment	5-Fluorouracil	hnRNPA2B1	2024	Zhang et al. (2022b)
	Binding proteins	Tumor promoter	Prognosis and Treatment	Cisplatin and Romidepsin	MSI2	2022	Liu et al. (2023)
m5C	Methyltransferase	Tumor promoter	Prognosis	—	NSUN2	2020	Yan et al. (2021)
​	Methyltransferase	Tumor promoter	Prognosis	—	NSUN2	2023	Liu S. et al. (2024)
​	Methyltransferase	Tumor promoter	Prognosis	—	NSUN2	2021	Li X. Y. et al. (2022)
​	Methyltransferase	Tumor promoter	Prognosis	—	NSUN2	2021	Christodoulidis et al. (2024)
​	Methyltransferase	Tumor promoter	Prognosis	—	NSUN5	2024	Yue et al. (2023)
​	Binding proteins	Tumor promoter	Prognosis and Treatment	5-Fluorouracil	YBX1	2025	Fatima (2025)
m7G	Methyltransferase	Tumor promoter	Prognosis	​	METTL1	2022	Pi et al. (2021)

## 4 Role of RNA methylation in drug resistance in GC

**FIGURE 3:**
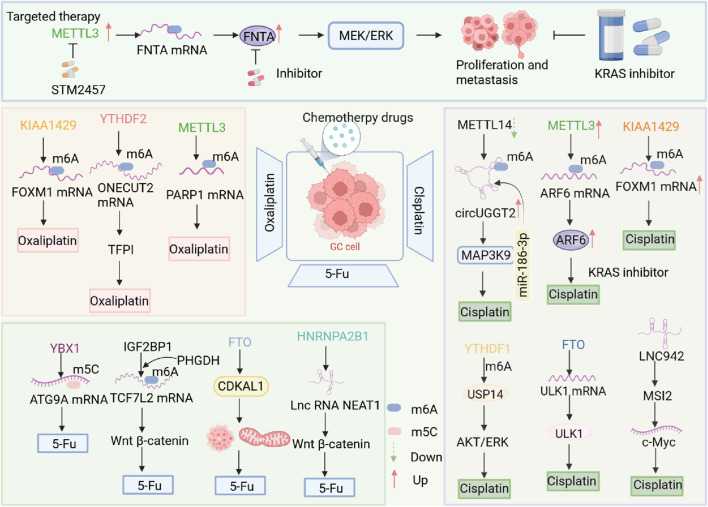
Abnormal RNA modification mechanisms involved in chemotherapy resistance in GC. Aberrant RNA methylation significantly enhances chemotherapy resistance in gastric cancer. KIAA1429, METTL3, and other regulators modulate m6A modifications, while YBX1 regulates m5C modifications, influencing drug sensitivity. Different RNA-modifying enzymes affect various RNAs, thereby regulating drug responses.

Tumor resistance is a significant challenge faced in cancer treatment, particularly during the treatment of gastric cancer, which significantly impacts patient prognosis and survival rates. Research shows that chemotherapy resistance is a major factor contributing to poor prognosis in gastric cancer patients, involving a variety of complex resistance mechanisms, including the tumor microenvironment, cancer stem cells, non-coding RNA, epigenetics, and epithelial-mesenchymal transition. As we’ve learned more about tumor biology in recent years, RNA methylation, an important epigenetic modification, has increasingly caught researchers’ attention for its role in tumor resistance. Studies show that RNA methylation plays a role in how gastric cancer cells resist treatment by regulating gene expression and cell signaling pathways (Figure 3). Current research suggests that changes in RNA methylation can influence tumor cell growth, death, and response to chemotherapy, indicating that it may play a key role in gastric cancer resistance. However, the exact ways RNA methylation impacts gastric cancer resistance are still unclear and need more investigation.

### 4.1 Alterations in biological cellular signaling pathways

Mechanisms of drug resistance in gastric cancer are closely related to changes in cell signaling. Research shows that RNA methylation can affect how gastric cancer cells resist chemotherapy by regulating the AKT/ERK and Wnt/β-catenin pathways, as well as the LNC942-MSI2-c-Myc axis. Specifically, hnRNPA2B1 stabilizes NEAT1 in a way that depends on m6A, which activates the Wnt/β-catenin signaling pathway to keep the CD133^+^/CD44^+^ stem cell phenotype, boosting resistance to 5-fluorouracil (Wang J. et al., 2024). In addition, RNA methylation is also key in how gastric cancer resists cisplatin. For example, YTHDF1 helps translate ubiquitin-specific peptidase (USP14) through an m6A-dependent pathway, activating the AKT/ERK pathway, which boosts the growth of gastric cancer cells and their resistance to cisplatin (Chen et al., 2022). LNC942 is highly expressed in drug-resistant gastric cancer cells and links to poor prognosis. It boosts MSI2 levels by blocking its degradation through ubiquitin, which in turn stabilizes c-Myc mRNA in an m6A-dependent way, helps prevent apoptosis, promotes stem cell traits, and boosts resistance to cisplatin in gastric cancer. Conversely, inhibiting the LNC942-MSI2-c-Myc axis may restore chemosensitivity, offering a new approach to treating drug-resistant gastric cancer (Zhu Y. et al., 2022). These changes in signaling pathways not only impact cancer cell growth and survival but might also increase their drug tolerance, which could affect how well treatments work in the clinic.

### 4.2 Gene expression and drug resistance

RNA methylation directly regulates gene expression by influencing RNA stability and translation efficiency. m6A, m5C, m7G and m1A can all modulate RNA stability and translation, and are closely related to the development and resistance of various cancers (Zhao et al., 2021). Among these, m6A and m5C are especially important in gastric cancer, which involves the dynamic regulation by methyltransferases, binding proteins, and demethylases. METTL3, METTL4, and KIAA1429 are the most common methyltransferases for m6A, playing a key role in regulating the expression of related genes in the context of gastric cancer resistance. Specifically, Li et al. demonstrated that m6A Methyltransferase METTL3 facilitates oxaliplatin resistance in CD133+ gastric cancer stem cells by Promoting PARP1 mRNA stability which increases base excision repair pathway activity (Li H. et al., 2022). Moreover, METTL3 increases ARF6 expression via m6A modification. This boosts gastric cancer cells’ resistance to cisplatin (Song et al., 2025). METTL14 inhibits the expression of circUGGT2 via m6A modification. CircUGGT2 is overexpressed in cisplatin-resistant gastric cancer cells and can bind competitively to miR-186-3p, lifting its inhibition of MAP3K9, which promotes the proliferation, invasion, and cisplatin resistance in gastric cancer cells (Chen X. Y. et al., 2024). Research shows that KIAA1429 is overexpressed in gastric cancer tissue samples, and its high levels of expression are linked to poor prognosis for gastric cancer patients. KIAA1429 enhances FOXM1 mRNA stability by targeting its m6A modification sites, thereby increasing gastric cancer cells’ resistance to oxaliplatin and cisplatin (Tang et al., 2023; Zhu Z. et al., 2022).

The m6A demethylation transferase and reading proteins also play a role in regulating drug resistance in gastric cancer cells. Studies have shown that FTO is overexpressed in gastric cancer and that its demethylase activity upregulates CDKAL1, which promotes cell proliferation and mitochondrial fusion, thereby increasing the resistance of gastric cancer to chemotherapy with 5-Fu (Liu et al., 2023). In addition, in cisplatin-resistant gastric cancer cells, m6A methylated RNA levels are significantly reduced, while FTO expression is increased. The study showed that blocking FTO reduces ULK1 via the m6A-YTHDF2 pathway, which blocks autophagy and ultimately reverses cisplatin resistance in SGC-7901/DDP cells (Zhang et al., 2022b). The m6A reader protein YTHDF2 mediates the m6A modification of ONECUT2 mRNA, thereby activating TFPI transcription, leading to stemness in gastric cancer and resistance to oxaliplatin, providing a new treatment target to tackle gastric cancer that is resistant to oxaliplatin (Fan et al., 2025).

In addition to the role of m6A methylation factors in gastric cancer resistance, m5C also plays a crucial role in how gastric cancer develops resistance. Specifically, the m5C reader protein YBX1 is overexpressed in gastric cancer cells and tissues resistant to 5-FU, and is linked to a poor prognosis. YBX1 stabilizes the ATG9A mRNA by modifying it with m5C, boosting autophagy, which ultimately helps gastric cancer cells resist 5-FU. Therefore, YBX1 is a key player in gastric cancer autophagy and resistance to 5-FU, and could be a potential target for overcoming resistance (Huang et al., 2025). The m5C methyltransferase NSUN2 is overexpressed in gastric cancer tissues and is linked to lymphatic metastasis and higher Ki67 expression. Downregulation of NSUN2 inhibits ERK1/2 phosphorylation, lowers the anti-apoptotic protein Bcl-2, and increases the pro-apoptotic protein Bax. This change boosts gastric cancer’s sensitivity to chemotherapy (Shen et al., 2024).

On the whole, RNA methylation has a significant impact on resistance to treatments in GC. The findings discussed above give us fresh insights into how tumors resist treatment and help us better understand the relevant biological foundations. Meanwhile, future studies can delve deeper into these mechanisms and may present prospective targets and approaches for tackling the issue of chemotherapy resistance in the management of GC.

## 5 RNA methylation mediates tumor immunity and resistance to targeted therapy

### 5.1 Regulatory role of RNA methylation in the immune system of tumors

The tumor immune microenvironment (TIME) represents a complex and ever-evolving ecosystem found within tumor tissues, consisting of various components, including immune cells, stromal cells, the extracellular matrix (ECM), soluble factors like cytokines and chemokines, as well as metabolic byproducts (Fatima, 2025). Recent research indicates that the RNA methylation is crucial for the regulation of immune responses in tumors: it impacts not only the functionality of immune cells but also the expression of immune-related factors by modulating RNA translation and stability; thereby, it emerges as a crucial contributor to the regulation of immune responses within tumors (Li Y. et al., 2024). Studies have indicated that the m6A modification plays a crucial role in modulating the expression of immune checkpoint proteins within tumor cells, and this regulation has an impact on T cell activity as well as the immune system’s response to tumors in the body (Li Y. et al., 2024). An illustrative example is KIAA1429, which serves as part of the MTC and exhibits elevated expression levels in hepatocellular carcinoma. This heightened expression level is linked to the increased levels of PD-L1, thereby facilitating the immune evasion of cancer cells (Jiang et al., 2025). Moreover, m6A modifications have the potential to regulate the infiltration of immune cells within the tumor microenvironment, thereby impacting the ability of the tumor to evade immune responses. This underscores that RNA methylation could represent a promising new target for immunotherapeutic strategies (Wu et al., 2024).

### 5.2 RNA methylation and the mechanism of tumor immune resistance

Research by Li et al. found that writers (such as METTL3 and METTL14) suppress T cell functions by enhancing the expression of immune checkpoints (PD-L1) and promoting glycolysis, leading to immunotherapy resistance; meanwhile, erasers (such as FTO and ALKBH5) maintain tumor stem cell characteristics and an immunosuppressive microenvironment by lowering m6A modification levels, weakening the effects of immunotherapy. Recognition proteins (such as YTHDF1 and IGF2BP3) promote immune evasion and resistance by stabilizing immune suppression-related mRNAs (such as PD-L1 and MYC) or inhibiting antigen presentation (such as MHC-I) (Li Y. et al., 2024).

Moreover, m5C regulatory proteins (such as NSUN2 and ALYREF) and m7G writers (such as METTL1) exacerbate resistance through metabolic reprogramming and the infiltration of immunosuppressive cells (such as MDSCs and Tregs). Targeting these regulatory proteins (such as METTL3 inhibitors STM2457 and FTO inhibitors FB23-2) can reverse resistance and enhance the efficacy of immune checkpoint blockade, providing a new approach to overcoming tumor immune resistance (Li Y. et al., 2024). Consequently, gaining a more profound insight into the mechanisms of how RNA methylation facilitates immune evasion will assist in the formulation of novel therapeutic approaches aimed at enhancing the effectiveness of tumor immunotherapy.

### 5.3 RNA methylation and mechanisms of resistance to targeted therapies

In the context of targeted therapy, the process of RNA methylation holds significant importance. METTL3 is overexpressed in GC tissues, enhancing FNTA translation through YTHDF1-dependent m6A modifications, maintaining KRAS membrane localization and continuously activating the MEK/ERK pathway, driving tumor progression and metastasis. Targeting METTL3 or FNTA can block this axis and restore sensitivity to KRAS inhibitors, providing new strategies to overcome targeted resistance in GC (Hu et al., 2025). In addition, the upregulation of METTL3 stabilizes HDGF mRNA through increased m6A modifications, enhancing tumor proliferation, metastasis and resistance to targeted drugs (Wang et al., 2020). High expression of FTO maintains the stability of resistance genes (such as MYC and β-catenin) through demethylation, leading to resistance to tyrosine kinase inhibitors (TKIs) and PARP inhibitors (Yan et al., 2018; Fukumoto et al., 2019); conversely, inhibiting METTL3 or FTO can reverse resistance and enhance sensitivity to targeted drugs and immunotherapy, making both key targets for overcoming targeted resistance in GC (Cui Y. H. et al., 2024). Studies have demonstrated that m6A modification plays a significant role in modulating the activity of the EGFR signaling pathway, thereby impacting tumor cell proliferation and their resistance to therapeutic agents. Specifically, the expression of METTL3 is upregulated in PLX4032-resistant melanoma cells, increasing m6A modification on EGFR mRNA, which in turn promotes EGFR protein translation efficiency, ultimately causing melanoma cells to become resistant to PLX4032 targeted therapy (Bhattarai et al., 2021). Furthermore, the level of METTL16 expression is linked to the resistance exhibited by tumor cells against targeted therapeutic interventions. METTL16 regulates the expression of PD-L1, thereby influencing immune evasion and drug resistance, which means that RNA methylation may alter the efficacy of targeted therapy through different pathways (Wang A. et al., 2023).

## 6 Conclusion

In recent years, RNA methylation has been recognized as a crucial regulatory mechanism for drug resistance in GC, hence its complexity and diversity have attracted significant attention from the scientific community. Through the investigation of different RNA modifications, researchers have progressively elucidated the functions of this epigenetic modification in the mechanisms underlying resistance to GC as well as the interconnected signaling pathways involved. Research in this field has not only provided new perspectives for understanding the mechanisms of GC drug resistance but also laid a theoretical foundation for improving clinical treatment strategies. However, there are certain discrepancies in the existing viewpoints and findings. On the one hand, some studies have emphasized the critical role of specific RNA methylation markers in the development of resistance; on the other hand, others have suggested that different environmental factors and genetic backgrounds may influence the patterns and effects of RNA methylation. Hence, an important direction for future research is to balance these different research results to form a more unified and comprehensive understanding of this field.

Future investigations utilizing single-cell level analyses have the potential to yield more nuanced insights into the dynamic alterations of RNA methylation. This approach will facilitate a better understanding of the intricate biological processes that occur within the tumor microenvironment. In addition, resistance prediction models based on RNA methylation characteristics can provide important references for the personalized treatment of GC patients. The clinical translation of these research findings is expected to promote the development and application of novel targeted drugs, thereby improving treatment outcomes and survival rates. To sum up, the importance of RNA methylation in the pathways associated with resistance to GC drugs is gaining prominence. However, there is currently a lack of research on how m1A methylation relates to drug resistance in GC; future studies could look into this matter and delve deeper into the potential clinical application of m1A levels as biomarkers for diagnosis and personalized therapeutic options. With the development of technology and more detailed research, we look forward to make breakthroughs in RNA methylation-targeted therapy in the near future, offering new hope to people facing GC.
